# The Combination of Soy Isoflavones and Resveratrol Preserve Bone Mineral Density in Hindlimb-Unloaded Mice

**DOI:** 10.3390/nu12072043

**Published:** 2020-07-09

**Authors:** Yuko Tousen, Ryota Ichimaru, Takashi Kondo, Masaki Inada, Chisato Miyaura, Yoshiko Ishimi

**Affiliations:** 1 Department of Food Function and Labeling, National Institute of Health and Nutrition, National Institutes of Biomedical Innovation, Health and Nutrition, 1-23-1 Toyama, Shinjuku-ku, Tokyo 162-8636, Japan; tousen@nibiohn.go.jp (Y.T.); s184757q@st.go.tuat.ac.jp (R.I.); t-condo@nibiohn.go.jp (T.K.); 2 Cooperative Major in Advanced Health Science, Tokyo University of Agriculture and Technology, 2-24-16 Nakamachi, Koganei, Tokyo 184-8588, Japan.; inada@cc.tuat.ac.jp (M.I.); miyaura@cc.tuat.ac.jp (C.M.); 3 Research Institute for Agricultural and Life Sciences, Tokyo University of Agriculture, 1-1-1 Sakuragaoka, Setagaya-ku, Tokyo 156-8502, Japan

**Keywords:** hindlimb-unloading, bone mineral density, soy isoflavones, resveratrol, bone strength

## Abstract

It is well known that physical inactivity during space flight or prolonged bed rest causes rapid bone loss. Soy isoflavones (ISOs) and resveratrol (RES) have been reported to be useful to maintain a positive balance for bone turnover. Therefore, we examined the combined effects of ISO and RES on bone loss that was induced by hindlimb-unloading in mice. Female eight-week-old ddY mice were divided into the following six groups (*n* = 6–8 each): normally housed mice, loading mice, hindlimb-unloading (UL) mice fed a control diet, UL mice fed a 0.16% ISO conjugates, UL mice fed a 0.15% RES diet, and UL mice fed a 0.16% ISO and 0.15% RES diet. After three weeks, femoral bone mineral density was markedly decreased in unloading mice. The combination of ISO and RES prevented bone loss and especially maintained the trabecular bone mineral density more effectively compared with cortical bones. ISO and/or RES inhibited the increase in the RANKL/OPG expression ratio in bone marrow cells in UL mice. These results suggest that the combination of ISO and RES had a preventive effect against bone loss induced by hindlimb-unloading in mice. These osteoprotective effects of ISO and RES may result from the inhibition of bone resorption.

## 1. Introduction

Osteoporosis, a major health problem, is an age-related disease that is characterised by bone mineralisation and microstructural declension, which raises the risk for bone fracture. Bone mass is influenced by genetic and environmental factors, such as mechanical loading, lifestyle, and nutrition. Among the environmental factors, mechanical loading and nutrition appear to be critical for prevention of osteoporosis. Skeletal unloading give rise a reduction in bone volume, mineralisation, and mechanical strength [1,2]. It has been reported that the older people and patients without weight-bearing activity exhibit reduced mechanical loading because increased bed rest and decreased physical activity promote bone loss [3,4]. Additionally, spaceflight raises rapid bone loss, especially in astronauts who are exposed to long-duration spaceflight, who lose a presumed 1.0%–1.6% of their bone mineral density (BMD) per month, which primarily arises in the cancellous compartments of the lower weight-bearing skeleton [5,6].

A hindlimb-unloaded model is widely accepted as an animal model for simulating and investigating weightlessness [7]. Mice or rats placed in a head-down tilt position and are suspended by their tails. Therefore, their hindlimbs, which are subjected to unloading, simulate microgravity and show a decrease in bone mass. This model results in decreased bone formation and increased bone resorption [8,9,10]. Thus, this model also induces a consequent loss of bone mass and decrease in bone mechanical strength [11]. To prevent bone loss induced by skeletal unloading, several different types of interventions including hormonal therapies and dietary compounds have been studied as countermeasures for bone loss related to skeletal unloading [8,12,13]. However, these drugs and compounds are sometimes associated with unfavourable outcomes, such as digestive disturbance, hepatic dysfunction, renal disturbance, diarrhoea, toxic epidermal necrolysis, and necrosis of the jawbone among others. Therefore, it would be ideal if functional foods were available to add to a diet. 

Some studies have reported that food components inhibit bone loss by mechanical unloading [14,15]. We have demonstrated that soy isoflavones (ISOs) and the combination of ISO and milk basic protein have prevented bone loss in hindlimb-unloading mice without adverse effects (e.g., diarrhoea and decreased plasma albumin concentrations, which reflect general health status) [16,17]. ISO is structurally similar to oestradiol, and it exhibits weak oestrogenic activity by binding to oestrogen receptors [18]. Numerous studies have demonstrated that ISO prevents bone loss in osteoporotic animal models [16,19].

Resveratrol (RES) is a natural polyphenol compound that is present in several plant foods; it is comparatively abundant in red grapes, berries, red wine, and peanuts, and it displays multiple biological activities [20]. RES belongs to the group of phytoestrogens, and it exhibited preventive effects for osteoporosis in in vitro and in vivo studies [21,22]. It was reported that RES promoted osteoblast differentiation and prevented osteoclast formation in vitro [21,23,24], attenuated ovariectomy (OVX)-induced bone loss, and increased bone strength in OVX rats [22,25]. Additionally, RES was reported to be protective against bone loss in hind-limb unloaded mature male rats [14,26]. Durbin et al. demonstrated that RES preserves bone mass, microstructure, and strength in hindlimb-unloaded old male rats [26]. Additionally, a protective role of RES on bone loss induced by unloading related to its anti-inflammatory effect was suggested. However, to the best of our knowledge, the effects of RES on bone using hind-limb unloaded female animals have not been investigated. 

Based on the evidence, we hypothesised that the combination of ISO and RES prevents bone loss caused by skeletal unloading. Therefore, in the present study, we examined the effects of the combination of ISO and RES on bone mineral density and mRNA expression of bone-related genes in bone marrow cells in hindlimb-unloaded mice.

## 2. Materials and Methods

### 2.1. Animals, Diets, and Experimental Design 

Female mice (ddY strain, 8 weeks) were purchased from Japan SLC (Shizuoka, Japan). Mice were housed in individual cages in a temperature- and humidity-controlled room (23 °C ± 1 °C and relative humidity of 60 ± 5%) with a 12-h light–dark cycle. After 7 days of acclimation, the mice were randomly divided into six body weight-matched groups: normally housed group (Normal: *n* = 6), loading group (Loading: *n* = 6), hindlimb-unloading mice fed a control diet (UL: *n* = 6), hindlimb-unloading mice that were fed a 0.16% ISO conjugate (Fujicco, Kobe, Japan) diet (UL-ISO: *n* = 8), hindlimb-unloading mice that were fed a 0.15% RES (purity: >99%; Nagara Science, Gifu, Japan ) diet (UL-RES: *n* = 8), and hindlimb-unloading group that were fed a 0.16% ISO conjugate and RES diet (UL-ISO + RES: *n* = 8). The normal group was not equipped for tail suspension, and were allowed to place their limbs on the cage bottom. Loading group was equipped for tail suspension, but the mice were allowed to place their limbs on the cage bottom. Hindlimb unloading groups (UL, UL-ISO, UL-RES and UL-ISO + RES groups) were subjected to unloading with the equipment for a tail suspension model [7]. Hindlimbs were always floated in unloading mice, but the forelimbs of the mice maintained contact with the cage bottom.

ISO (Fujiflavone P40: ISO content, 40%) is a soy ISO conjugate, with the following aglycones in 100 mg of conjugates: 33 mg of daidzein, 8.5 mg of genistein, and 15 mg of glycitein. The mice were fed an AIN-93G [27] diet with corn oil instead of soybean oil with or without 0.16% ISO conjugates and/or 0.15% RES for 3 weeks (Table 1). ISO conjugates was mixed with the powder diet. We added water to the powder diet. The mice were fed the solid diet. The ISO concentration was chosen based on the results of previous studies. Regarding to the results of our previous studies, bone loss was significantly inhibited by 0.20% ISO conjugate treatment in hindlimb unloading mice [17]. Although the dose of ISO used in this study was relatively higher than other nutritional experiments, we chose 0.16% ISO conjugate in order to assess the combination of ISO and RES on bone loss. The sample size used in our study was determined based on the results of a previous study [28]. The sample size calculation was performed before the start of the study and indicated that 5 mice per group would show a difference of 6.2 mg/cm^2^ between femur BMD means, assuming a standard deviation (SD) of 2.6 mg/cm^2^, with power of 0.80 and a two-sided type 1 error of 0.05. Therefore, 6-8 mice in each group was considered an appropriate sample size for the study.

The mice were pair-fed their respective diets, with free access to distilled water during this period. The amount of food intake was weighed during the experimental period. The mice were fasted overnight before dissection, and euthanised by exsanguination under anaesthesia. Blood was then collected in vacutainers and centrifuged at 700× *g* at 4 °C for 15 min. The plasma was removed and stored at −80 °C until it was assayed. The left femur was removed, submerged in 70% ethanol, and stored at 4 °C until BMD measurement. The right tibia was removed to extract total RNA from the bone marrow cells. For histological analyses, the right femur was removed and stored in 70% ethanol at 4 °C. All procedures involving animals were in accordance with the guidelines for the care and use of laboratory animals by the National Institute of Biomedical Innovation, Health and Nutrition (Tokyo, Japan) and the ethics committee approved the study protocol (DS28-59, 31 March 2017). 

### 2.2. Microcomputed Tomography (μCT) Analysis of the Distal Femur

Distal femurs were scanned at 48-μm intervals using an experimental animal CT system (LaTheta LCT-200; Hitachi Aloka Medical, Tokyo, Japan). Analyses of distal femurs were performed in a region of the trabecular and cortical bone from the end of distal femur to the growth plate extending 7.5 mm towards the diaphysis. The minimum moment of inertia of cross-sectional areas (MMICA) and the polar moment of inertia of cross-sectional areas (PMICA) were calculated using LaTheta software (ver. 1.31; Hitachi Aloka Medical, Tokyo, Japan).

### 2.3. Bone Histological Stain Analysis

Right femurs were embedded in glycolmethacrylate without decalcification. Then, serial sections (3-µm thick) were cut longitudinally using a microtome (model 2255; Leica Microsystems, Wetzlar, Germany). Sections were stained with Villanueva Goldner stain to distinguish between mineralised and unmineralised bone and identify cellular components. In Villanueva Goldner-stained sections, calcified bone presented as green and osteoids presented as red. Histological analyses were conducted by Kureha Special Laboratory (Fukushima, Japan).

### 2.4. Analysis of Plasma Albumin and Total Protein Concentrations

Plasma albumin and protein concentrations were measured by A/G B-test Wako (FUJIFILM Wako Pure Chemical, Osaka, Japan), in accordance with the manufacturer’s instructions.

### 2.5. RNA Extraction and Quantitative Real-time PCR 

Total RNA was extracted from the bone marrow of the tibia using Isogen® II, in accordance with the manufacturer’s (NIPPON GENE, Tokyo, Japan) instructions. Complementary DNA (cDNA) was synthesised from 1 μg of total RNA using PrimeScript™ RT Master Mix (TaKaRa Bio, Shiga, Japan). cDNA was quantified by real-time PCR using SYBR™ Premix Ex Taq II (TaKaRa Bio, Shiga, Japan). PCR conditions were 95 °C for 30 s, followed by 40 cycles of 95 °C for 5 s and 60 °C for 30 s. The primer sequences are shown in Supplementary Table S1. The results from bone marrow cells are expressed as the fold-change relative to those of normal mice after normalisation to beta-actin (*Actb*) gene expression.

### 2.6. Statistical Analysis

Data are presented as the mean ± standard error of the mean (SEM). The data were analysed using one-way analysis of variance (ANOVA). Differences among the groups were assessed using Tukey’s post hoc test. If variances were not equivalent, the data were analysed using a Kruskal–Wallis ANOVA, and followed by a Steel-Dwass test. Differences were considered to be significant if *P* < 0.05. Statistical analyses were undertaken using SPSS v19 (IBM, Armonk, NY, USA). The normal group was not included for statistical analysis in this study. Because the main objective in this study was to determine the effects of the combination of ISO and RES on bone loss induced by hindlimb unloading in mice, the loading group was used as the control group.

## 3. Results

### 3.1. Body Weight, Food Intake, and Plasma Albumin and Total Protein Concentrations

**Figure 1:**
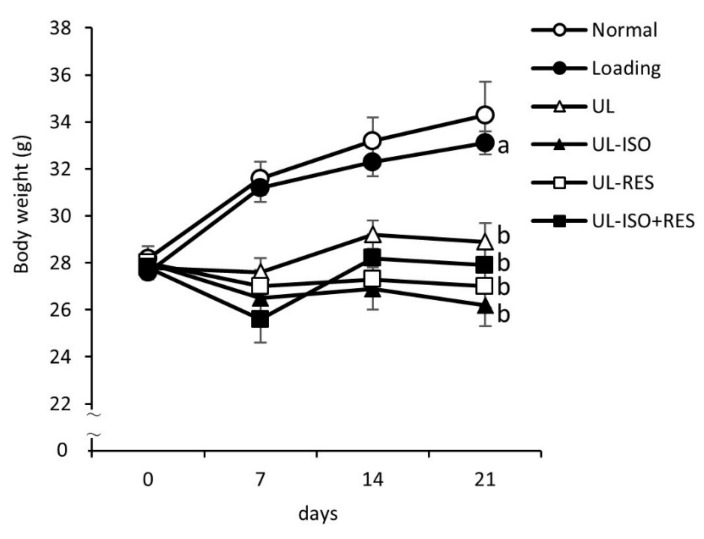
Body weight in hindlimb-unloading mice. Normal, normally housed group; Loading, loading group; UL, hindlimb-unloading mice fed a control diet; UL-ISO, hindlimb-unloading mice fed a 0.16% soy-isoflavones conjugates diet; UL-RES, hindlimb-unloading mice fed a 0.15% RES diet; UL-ISO + RES; hindlimb-unloading mice fed a 0.16% soy-isoflavones conjugates and resveratrol diet. Values are the mean ± SEM (*n* = 6–8). The data were analysed using one-way analysis of variance (ANOVA). The Normal group was not included in the statistical analyses. Differences among the groups were assessed by Tukey’s post hoc test. If variances were not equivalent, the data analysed Kruskal–Wallis ANOVA, and followed by a Steel-Dwass test. Differences were considered significant when *P* < 0.05. ^a, b^ Mean values with unlike letters were significantly different.

Initial body weight did not differ significantly among all groups (Table 1 and Figure 1). However, the final body weight from the UL groups was significantly lower than that in the Loading group (Table 2 and Figure 1). Treatment with ISO or/and RES did not affect body weight in UL mice. Significant differences in total food intake were not observed among groups (Table 1). There were no differences in the concentrations of plasma albumin and total protein among the groups (Table 1).

### 3.2. Femur BMD and Bone Strength by uCT

**Figure 2:**
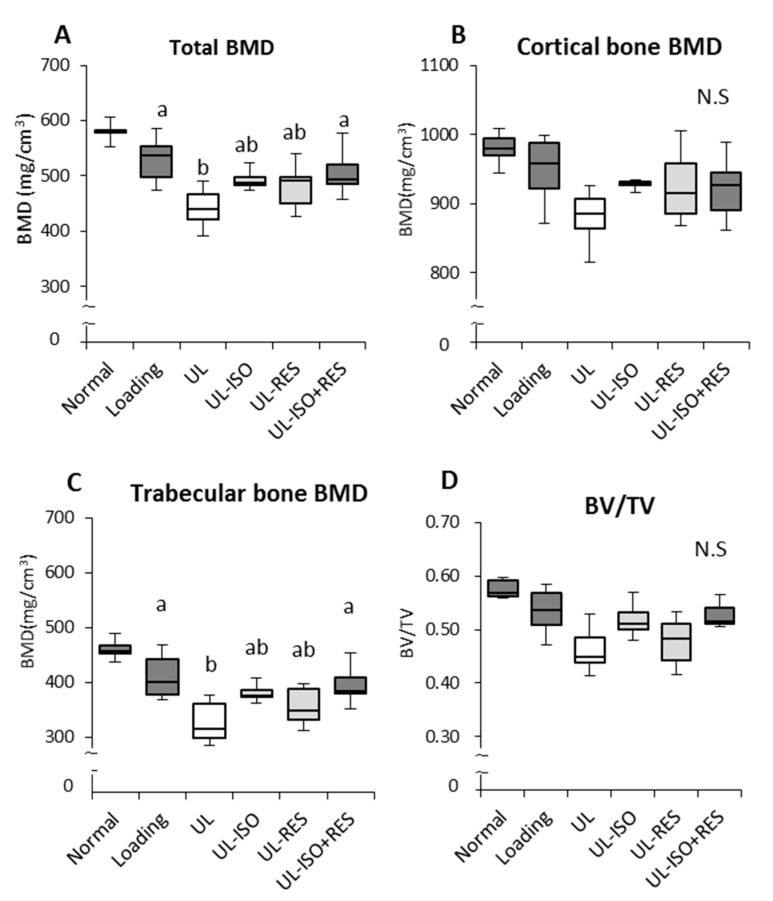
Bone mineral density (BMD) and bone volume per tissue volume (BV/TV) in femur in hindlimb-unloading mice. Normal, normally housed group; Loading, loading group; UL, hindlimb-unloading mice fed a control diet; UL-ISO, hindlimb-unloading mice fed a 0.16% soy-isoflavones conjugates diet; UL-RES, hindlimb-unloading mice fed a 0.15% RES diet; UL-ISO + RES; hindlimb-unloading mice fed a 0.16% soy-isoflavones conjugates and resveratrol diet. BMD of (**A**) the total, (**B**) Cortical and (**C**) trabecular bone on distal femur, and (**D**) bone volume per tissue volume (BV/TV) were analysed by micro-computed tomography scanning. Values are the mean ± SEM (*n* = 6–8). The data were analysed using one-way analysis of variance (ANOVA). The Normal group was not included in the statistical analyses. Differences among the groups were assessed by Tukey’s post hoc test. If variances were not equivalent, the data analysed Kruskal–Wallis ANOVA, and followed by a Steel-Dwass test. Differences were considered significant when *P* < 0.05. ^a, b^ Mean values with unlike letters were significantly different.

Hindlimb unloading caused a significant decrease in femoral total BMD and trabecular bone BMD (Figure 2A,C). Cortical bone BMD and BV/TV tended to be lower in the UL group compared with the Loading group (Loading vs. UL; *P* = 0.079 and *P* = 0.052, respectively) (Figure 2B,D). The decrease was more pronounced in the trabecular bone BMD than in cortical bone BMD. The combination of ISO + RES treatment significantly attenuated total bone loss (UL vs. ISO + RES; P = 0.038, 16% decrease vs. 4% decrease) and trabecular bone loss (UL vs. ISO + RES; *P* = 0.013, 20% decrease vs. 4% decrease) (Figure 2A,C). However, there was no significant difference between the UL-ISO or the UL-RES group and the UL group (Figure 2A,C). The combination of ISO + RES tended to prevent the decrease in the BV/TV (UL vs. ISO + RES; *P* = 0.059, 13% decrease vs. 1% decrease) induced by unloading (Figure 2D). However, cortical bone BMD was not significantly affected by unloading, ISO, and/or RES (Figure 2B). 

**Figure 3:**
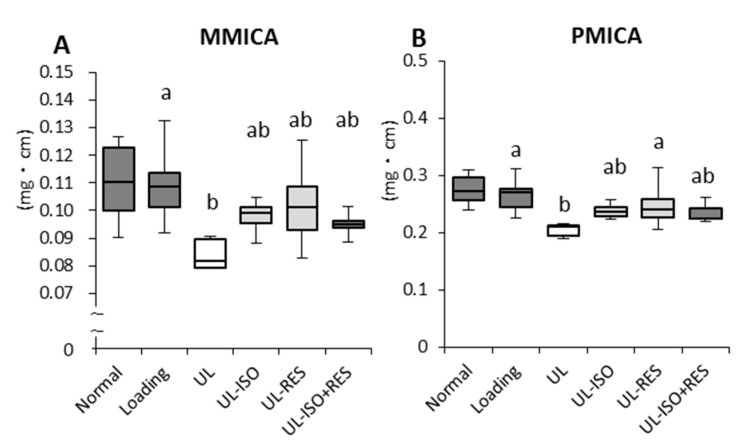
Bone strengths in femur in hindlimb-unloading mice. Bone strength parameters were measured by micro-computed tomography scanning. (**A**) Minimum moment of inertia of cross-sectional areas (MMICA). (**B**) Polar moment of inertia of cross-sectional areas (PMICA). Normal, normally housed group; Loading, loading group; UL, hindlimb-unloading mice fed a control diet; UL-ISO, hindlimb-unloading mice fed a 0.16% soy-isoflavones conjugates diet; UL-RES, hindlimb-unloading mice fed a 0.15% RES diet; UL-ISO + RES; hindlimb-unloading mice fed a 0.16% soy-isoflavones conjugates and resveratrol diet. Values are the mean ± SEM (n = 6–8). The data were analysed using one-way analysis of variance (ANOVA). The Normal group was not included in the statistical analyses. Differences among the groups were assessed by Tukey’s post hoc test. If variances were not equivalent, the data analysed Kruskal–Wallis ANOVA, and followed by a Steel-Dwass test. Differences were considered significant when *P* < 0.05. ^a, b^ Mean values with unlike letters were significantly different.

To confirm the effects of ISO and/or RES treatment on the bone strength of the distal femur in unloading mice, bone morphometric analyses were performed using microcomputed tomography. Hindlimb unloading caused a significant decrease in MMICA and PMICA, which are parameters of bone strength (Loading vs. UL; *P* = 0.048 and *P* = 0.004, respectively) (Figure 3A,B). The decrease in MMICA and PMICA caused by unloading was slightly lowered by ISO or ISO + RES treatment, but this was not significant (Figure 3A,B). RES treatment significantly attenuated the PMICA of the distal femur (Figure 3B). 

### 3.3. Bone Histological Stain Analysis

**Figure 4:**
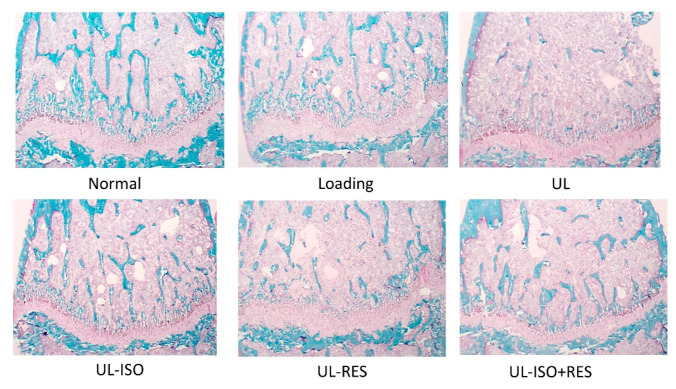
Bone histological stain analysis of distal femur in hindlimb-unloading mice. The right femurs were fixed, and embedded in glycolmethacrylate without decalcification. Then, serial sections were cut longitudinally, and sections were stained with Villanueva Goldner stain for discrimination between mineralized and unmineralized bone, and for the identification of cellular components. In Villanueva Goldner-stained sections, calcified bone is represented in green and osteoids are represented in red. Normal, normally housed group; Loading, loading group; UL, hindlimb-unloading mice fed a control diet; UL-ISO, hindlimb-unloading group fed a 0.16% soy-isoflavones conjugates diet; UL-RES, hindlimb-unloading group fed a 0.15% RES diet; UL-ISO + RES; hindlimb-unloading group fed a 0.16% soy-isoflavones conjugates and resveratrol diet.

Figure 4 shows histological staining of distal femurs in each group. There were markedly fewer blue-stained areas representing calcified bone in the distal femur in the UL group compared with those in the loading group. The ISO and/or RES treatment slightly inhibited this change (Figure 4).

### 3.4. Quantitation of mRNA Expression in Bone Marrow Cells from the Tibia

**Figure 5:**
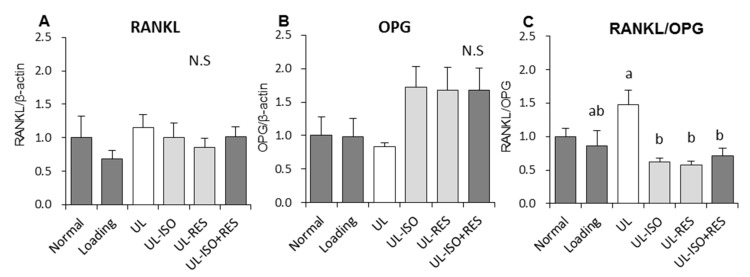
Bone metabolism-related gene expressions in bone marrow cells collected from in hindlimb- unloading mice. Expression levels receptor activator of nuclear factor kappa-B ligand (RANKL) and osteoprotegerin (OPG) were determined by quantitative real-time PCR. The ordinate axis indicates the relative amount of mRNA compared with Normal mice. Gene expression levels were normalized with β-actin. (**A**) RANKL. (**B**) OPG. (**C**) RANKL/OPG ratio. Normal, normally housed group; Loading, loading group; UL, hindlimb-unloading mice fed a control diet; UL-ISO, hindlimb-unloading mice fed a 0.16% soy-isoflavones conjugates diet; UL-RES, hindlimb-unloading mice fed a 0.15% RES diet; UL-ISO + RES; hindlimb-unloading mice fed a 0.16% soy-isoflavones conjugates and resveratrol diet. Values are the mean ± SEM (*n* = 6–8). The data were analysed using one-way analysis of variance (ANOVA). The Normal group was not included in the statistical analyses. Differences among the groups were assessed by Tukey’s post hoc test. If variances were not equivalent, the data analysed Kruskal–Wallis ANOVA, and followed by a Steel-Dwass test. Differences were considered significant when *P* < 0.05. ^a, b^ Mean values with unlike letters were significantly different.

We investigated the effect of ISO and/or RES on bone metabolism-related gene expression in bone marrow cells. There was no significant difference in the mRNA expression of a receptor activator of nuclear factor kappa-B ligand (RANKL) and osteoprotegerin (OPG) among the groups (Figure 5A,B). The ratio of RANKL/OPG in the UL group tended to be higher than that in the loading group (*P* = 0.064) (Figure 5C). ISO and/or RES treatment significantly inhibited the increase in the ratio that was caused by unloading, however a significant difference was not observed between ISO or RES groups and the ISO+RES group.

## 4. Discussion

The present study demonstrated that the combination of ISO and RES treatment for 3 weeks prevented the decrease in femur BMD that was caused by hindlimb-unloading in female mice without adverse effects. The ISO and/or RES treatment also inhibited the increased the RANKL/OPG mRNA expression ratio, which is the critical bone resorption factor that is caused by unloading in bone marrow cells in unloading mice. 

Our results showed that the decrease in BMD caused by unloading was significantly prevented by the combination of 0.16% ISO conjugates and 0.15% RES treatment (Figure 2A). However, although the decrease in total bone and trabecular bone BMDs caused by unloading was slightly attenuated by ISO or RES treatment, there was no significant difference between the UL-ISO or the UL-RES and the loading groups. This was especially observed in mice that were fed ISO and/or RES that were maintained on their trabecular BMD more effectively compared with cortical bones (Figure 2B,C). A reduction in BV/TV indicates a reduction in cancellous bone volume. Although there was no statistically significant difference in BV/TV, the combination of ISO and RES treatments tended to prevent the reduction in BV/TV in the femur (*P* = 0.0592) (Figure 2D). Moreover, bone histological stain analysis of the distal femur that was observed for ISO and/or RES treatment inhibited the reduction in calcified bone that was caused by unloading (Figure 4). Trabecular bone is also more responsive than cortical bone for mechanical unloading. Previously, we reported that the combination of 0.2% ISO conjugates and 1.0% milk basic protein significantly inhibited the distal bone loss in the femur in hindlimb-unloaded female mice [17]. The distal femur is subject to bone resorption, because the femur is rich in trabecular bone, which has a large surface. However, there was no significant effect of the combination of ISO conjugates and BMP in the middle femoral BMD, which is rich in cortical bone [17], and this is also consistent with the results of this study. Based on these findings, the combination of ISO and RES treatment prevents unloading-induced reduction in BMD, and it might be especially effective for trabecular bone in unloading mice. 

In previous studies, we demonstrated that a 0.20% or 0.25% ISO conjugate-containing diet (ISO 320 or 400 mg/kg body weight (BW)/day) significantly prevented unloading-induced bone loss in hindlimb-unloading mice [16,17]. In the present study, to focus on the combined effect of ISO and RES, the ISO dose (0.16% ISO diet; ISO 256 mg/kg BW/day) was decreased compared with that in our previous studies. Thus, in the present study, the effect of ISO on bone loss was milder than that in our previous studies. Some studies support the bone-sparing effect of RES in hindlimb-unloaded animal models [26,29,30]. Habold et al. reported that RES at a dose of 400 mg/kg/day orally for 45 days before and during the 14 days of hindlimb unloading prevented microarchitecture deterioration in the long bones in hindlimb-unloaded mature male rats [29]. Durbin et al. reported that providing resveratrol at a dose of 12.5 mg/kg BW/day by oral gavage for 21 days preserved long bone mass, microstructure, and bone strength in hindlimb-unloaded old male rats [26]. The RES treatment dose in our study (240 mg/kg BW/day) is the same dose as in previous studies [26,29,30]. However, there was a difference in the administration methods, the dose period, sex, and age between this study and previous studies [26,29,30]. The observation of no significant effect in our study might be because of these differences. In this study, ISO and/or RES were mixed in the diet in order to extrapolate the method of eating in humans. When ISO and/or RES was mixed with the diet, these compounds were better able to persist their concentrations in the blood and for metabolism compared with that with simply oral administration. The hindlimb-unloaded animal model is almost exclusively a male model. Conversely, it is reported that many food components effective for bone loss have weak estrogenic effects, such as ISO and RES. Thus, it is necessary to evaluate the effects of compounds on females, including their adverse effects. Additionally, bone strength is related to the integration of two main features: BMD and bone quality. Our data showed that the parameters of bone strength were significantly decreased, which was caused by unloading, and ISO and/or RES treatment ameliorated the unload-induced decrease. Durbin et al. reported that RES treatment ameliorated unload-induced loss of femoral strength resulting from the protective effect on femoral BMD and bone microarchitecture [26]. Moreover, several experimental studies demonstrated that ISO significantly inhibited a reduction of BMD, breaking strength, and bone quality in OVX rats [31,32]. These results suggest that the combination of ISO and RES prevent unloading-induced trabecular bone loss with consequent ameliorative effects on bone strength. Hindlimb-unloading for three weeks did not show a reduction in BMD or in the bone strength parameters for the humerus (Supplementary Figure S1). This result is consistent with previous study, which suggested that BMD in the loading site was not affected by tail suspension. 

The reduction in bone mass that was induced by skeletal unloading has been reported in many studies [7,8,9,10]. Studies showed that bone loss is associated with both the increase in bone resorption and the decrease in bone formation [8,9,10,32]. In other studies, for unloading-induced bone loss in rats, suppression of bone formation played a predominant role rather than acceleration of bone resorption [10,11,30]. Thus, the unloading model reflects an imbalance in bone remodelling. In the present study, we observed that ISO and/or RES treatments inhibited the increase in the RANKL/OPG mRNA expression ratio that was caused by unloading (Figure 5C). Activated RANKL related to RANK, which excites on the osteoclasts for bone resorption. OPG is an anti-resorptive agent that acts as a decoy receptor for RANKL [33]. Inhibition of the RANKL/RANK pathway supresses osteoclast formation, differentiation, and activation and bone resorption [33]. Thus, the RANKL/OPG ratio represents a critical role in bone resorption. It was reported that ISO treatment decreased the serum RANKL/OPG balance in OVX rats [31]. In addition, RES was shown to inhibit RANKL/OPG expression in the OVX rats femur [22]. These results suggest that ISO and/or RES prevent bone resorption caused by hindlimb-unloading through regulating RANKL and OPG mRNA expression in bone marrow cells. However, further studies are needed to elucidate the precise mechanisms through which ISO and/or RES attenuate unloading-induced bone loss.

In the present study, there were no adverse effects of ISO and/or RES supplementation. The unloading mice in this study seemed to be in a normal condition during the study because the plasma albumin and total protein concentrations (Table 2), which partially reflect the general health condition of the mice. The level in the UL groups were not lower than that in the loading group. Additionally, we measured the organs weight at the dissection, there were no effects of hindlimb unloading and ISO and/or RES treatments. The limitation of this study is that hindlimb unloading in mice causes a persistent reduction in body weight when compared with that in the loading group. The reduction in body weight might be a result of persistent endocrine stress. Hindlimb-unloaded mice are used as an animal model for weightlessness, such as for subjects with disabilities as well as astronauts. This animal model helped obtain scientific evidence, which is not possible in humans. However, this is a pre-clinical study, and data in mice cannot necessarily be extrapolated to humans. In this study, the doses in the diets were ISO 13.6 mg/kg BW (aglycone equivalent) and RES 20.5 mg/kg BW, which were calculated using the body surface area normalization method [34]. This dose is too high and is not reasonable for the amount one would get from eating a normal diet. Moreover, the metabolic rate is faster in mice than humans. In this study we did not measure plasma ISO concentration, but in our previous study of the level of plasma ISO, the mice fed an ISO diet were almost the same as those in the previous reports that revealed bone sparing effects of ISO in rodents and humans [16,17,35,36,37,38].

## 5. Conclusions

We found that the combination of ISO and RES treatment prevents bone loss and decreases the RANKL/OPG gene expression ratio in bone marrow cells in unloading mice. These results suggest that the combination of ISO and RES had a preventive effect against bone loss in total and trabecular bone of femur induced by hindlimb unloading in mice. These osteoprotective effects of the combination of ISO and RES may, in part, result from the inhibition of bone resorption. Further studies will be required to clarify the mechanisms underlying the osteoprotective effects of ISO and RES treatment in the hindlimb unloading condition.

## Figures and Tables

**Table 1:** Composition of the experimental diets^a^.

Ingredient (g)	Normal ^b^, Loading ^b^	ISO ^c^	RES ^d^	ISO + RES ^e^
Cornstarch	529.5	525.5	528.0	524.0
Casein	200	200	200	200
Sucrose	100	100	100	100
Corn oil	70	70	70.0	70
Cellulose	50	50	50.0	50
Mineral mixture ^a^	35.0	35.0	35.0	35.0
Vitamin mixture ^a^	10.0	10.0	10.0	10.0
L-Cystine	3.00	3.00	3.00	3.00
Choline bitartrate	2.50	2.50	2.50	2.50
Tert-Butylhydroquinone	0.014	0.014	0.014	0.014
Isoflavones ^f^	-	4.00	-	4.00
Resveratrol ^g^		-	1.50	1.50
Total	1000	1000	1000	1000

^a^ Prepared according to the AIN-93G formulation [27]. ^b^ Control diet. ^c^ Isoflavone-supplemented diets (ISO). ^d^ Resveratrol-supplemented diets (RES). ^e^ Isoflavone and resveratrol-supplemented diets (ISO + RES). ^f^ Isoflavones (Fujiflavone P40: ISO content, 40%) is an ISO conjugates, with the following aglycones in 100mg of conjugates: 33mg daidzein, 8.5 mg genistein, and 15 mg glycitein. ^g^ Resveratrol is purity: >99%; Nagara Science Co., Ltd., Gifu, Japan.

**Table 2:** Body weights, food intake, and plasma total protein and albumin concentration in mice.

	Normal	Loading	UL	UL- ISO	UL- RES	UL- ISO＋RES	*p*
Body weight							
Initial body weight (g)	28.2 ± 0.5	27.6 ± 0.3	27.8 ± 0.4	28.0 ± 0.4	28.0 ± 0.4	27.8 ± 0.4	0.925
Final body weight (g)	31.9 ± 1.5	31.2 ± 0.5 ^a^	27.0 ± 0.7 ^b^	24.7 ± 0.8 ^b^	25.2 ± 0.8 ^b^	26.1 ± 0.7 ^b^	< 0.01
Total food intake (g)	88.4 ± 0.7	88.8 ± 1.4	87.8 ± 1.5	85.2 ± 1.2	86.3 ± 1.7	84.0 ± 1.9	0.200
Plasma							
Total protein (g/dL)	4.19 ± 0.15	4.45 ± 0.10	4.62 ± 0.17	4.47 ± 0.14	4.61 ± 0.09	4.59 ± 0.05	0.733
Albumin (g/dL)	2.48 ± 0.10	2.74 ± 0.12	2.59 ± 0.11	2.57 ± 0.12	2.87 ± 0.07	2.84 ± 0.05	0.091

Normal, normally housed group; Loading, loading group; UL, hindlimb-unloading mice fed a control diet; UL-ISO, hindlimb-unloading mice fed a 0.16% soy-isoflavones conjugates diet; UL-RES, hindlimb-unloading mice fed a 0.15% RES diet; UL-ISO + RES; hindlimb-unloading mice fed a 0.16% soy-isoflavones conjugates and resveratrol diet. Values are the mean ± SEM (*n* = 6–8). The data were analysed using one-way analysis of variance (ANOVA). The Normal group was not included in the statistical analyses. Differences among the groups were assessed by Tukey’s post hoc test. If variances were not equivalent, the data analysed Kruskal–Wallis ANOVA, and followed by a Steel-Dwass test. Differences were considered significant when *P* < 0.05. ^a, b^ Mean values with unlike letters were significantly different.

## Supplementary Materials

The following are available online at https://www.mdpi.com/2072-6643/12/7/2043/s1: Figure S1, Bone mineral density (BMD), bone volume per tissue volume (BV/TV), and bone strengths parameters in humerus in hindlimb unloading mice; Table S1, Sequence of primers used for quantitative real-time PCR.
