# Supplementary material for: The Combination of Soy Isoflavones and Resveratrol Preserve Bone Mineral Density in Hindlimb-Unloaded Mice
Source: Nutrients. 2020 Jul 9;12(7):2043. doi: 10.3390/nu12072043 (PMC7400925; doi:10.3390/nu12072043)
Supplement: Supplementary file 1 [file nutrients-12-02043-s001.zip › Proof supplementary files/ISO+RES Supplementary Table S1.docx]

**Table S1.** Sequence of primers used for quantitative real-time PCR.

|  | Protein (gene) | Forward primer (5’ to 3’) | Reverse primer (5’ to 3’) |
| --- | --- | --- | --- |
|  | β-actin (*Actb*) | 5′-CCACAGCTGAGAGGGAAATC-3′ | 5′-AAGGAAGGCTGGAAAAGAGC-3’ |
|  | RANKL^a^ (Tnfsf11) | 5'-TGAAGACACACTACCTGACTCCTG -3’ | 5’-CCACAATGTGTTGCAGTTCC-3’ |
|  | OPG^b^ (Tnfrsf11b) | 5'-TCCTGGCACCTACCTAAAACAGCA-3’ | 5'-ACACTGGGCTGCAATACACA-3’ |

^a^ Receptor activator of nuclear factor kappa-B ligand, ^b^Osteoprotegerin
