# Supplementary figures and images for: The Combination of Soy Isoflavones and Resveratrol Preserve Bone Mineral Density in Hindlimb-Unloaded Mice
Source: Nutrients. 2020 Jul 9;12(7):2043. doi: 10.3390/nu12072043 (PMC7400925; doi:10.3390/nu12072043)

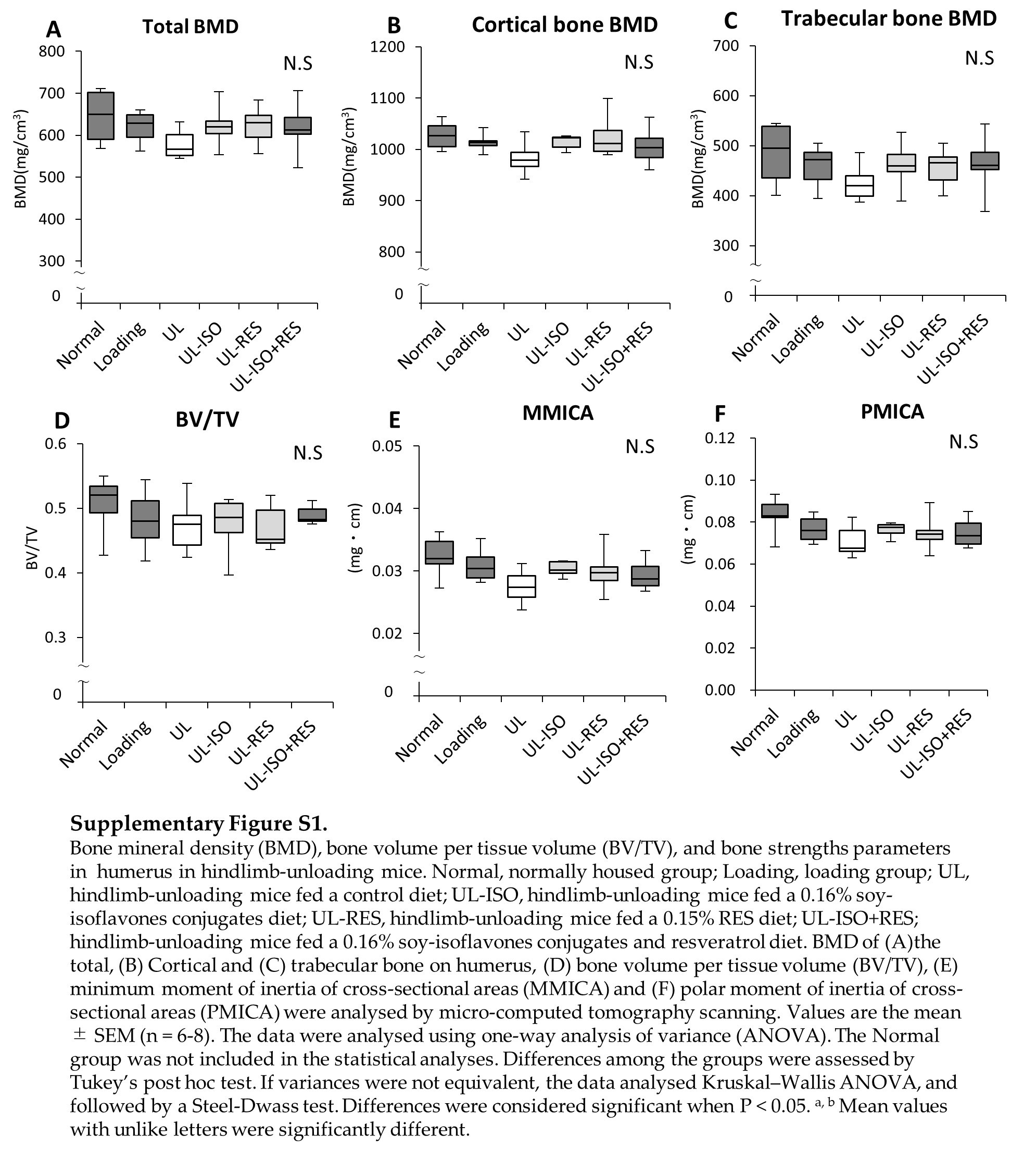

Supplement: Supplementary file 1 [file nutrients-12-02043-s001.zip › Proof supplementary files/ISO+RES_Supplementary_Figure. S1.jpg]
